# Molecular docking analysis of bioactive compounds from Mollugo cerviana (L.) SER with DHFR for antifungal activity

**DOI:** 10.6026/97320630017944

**Published:** 2021-11-30

**Authors:** F Sherin Rebecca, Rajamanickam Pon Nivedha, R Sharmila

**Affiliations:** 1 Department of Zoology, Jayaraj Annapackiam College for Women (Autonomous), Periyakulam, India; 2 Department of Biotechnology, Holy Cross College (Autonomous), Tiruchirappalli, India; 3 Department of Biotechnology, Bishop Heber College, Tiruchirappalli, India

**Keywords:** Aspergillus niger, Candida albicans, Mollugo cerviana, DHFR, Molecular docking

## Abstract

Fungal infections have been increasing in recent years due to growing number of high-risk patients particularly immuno compromised hosts. Candida is the third- or fourth-most-common isolate in nosocomial bloodstream infections. The increase of fungal resistance to classical drugs, the treatment costs, and the fact that most available antifungal drugs have only fungistatic activity, justify the search for new strategies. Identification of therapeutic compounds from plants has been the centre of attraction ever since they were discovered. It is of interest to document the molecular docking analysis of bioactive compounds present in Mollugo cerviana (L.) SER with the DHFR protein target for antifungal activity. We show the optimal binding features of several compounds from the extract with in vivo and in vitro activities. Results of this showed that all compounds showed good antimicrobial activity and a very good antifungal activity against the target DHFR protein. So, these compounds may act as potential drug molecules after the experimental validation.

## Background:

Fungal infection reports more than 1.5 million deaths annually worldwide [1]. Modern medicine provides many new prophylactic antifungal drugs to treat these infections. Despite the use of these drugs, the efficacy and therapeutic ability are still been limited due to their increased toxicity level and development of resistance towards these antifungal drugs [2]. The antifungal properties of many new plant extracts with high efficiency have been reported [3,4]. The chosen medicinal plant Mollugo cerviana (L.) SER is loaded with large number of phytochemicals and reported for its antibacterial, anifungal and anti-inflammatory activity [5]. This plant is used as a traditional medicine in south Indian Villages to treat fever, Stomach ache, Jaundice, improving eye sight and to regulate Blood Pressure and they exhibit good hepato protective efficiency [6]. Therefore, it is of interest to document the molecular docking analysis of these bioactive compounds in Mollugo cerviana (L.) SER with the DHFR protein target for antifungal activity.

## Materials & Methods:

### Plant material:

The aerial plant of Mollugo cerviana was collected near Sathankulam, Thoothukudi district, Tamil Nadu, India. The plant was botanically identified and authenticated by Dr.V. Chelladurai, Research Officer (Retired), Botany, Central Council for Research in Ayurvedic Sciences, Govt. of India and the specimen was deposited at department of botany, Auxilium College, Vellore.

### Preparation of plant extract:

The aerial parts of plants were air dried and powdered mechanically. About 500 g of the plant powder were extracted by successive extraction method using Methanol. The extracts obtained were filtered and evaporated on water bath to get crude extracts. These extracts were used for the further studies.

### Antifungal activity:

Microorganism used:

The in vitro antifungal strain such as Aspergillus niger ATCC 9029 and Candida albicans ATCC 2091 were used for present studies and these microbes were obtained from microbial type collection centre, Chandigarh, India.

### Paper disc diffusion method:

The sterilized (autoclaved at 121°C for 15 min) medium (40‐50°C) was inoculated (1 ml/100 ml of medium) with the suspension (105 cfu/ml) of the microorganism (matched to McFarland barium sulphate standard) and poured into a petridish to give a depth of 3-4 mm. The paper impregnated with the methanolic extract (25, 50 and 100 µg/ml in dimethyl formamide) was placed on the solidified medium. The plates were preincubated for 1 hr at room temperature and incubated at 37°C for 48 hrs and antifungal activity were recorded. Ketoconazole (50 µg/disc) was used as standard for antifungal activity [7].

### Minimum inhibitory concentration (MIC):

MIC of the extract was determined by agar streak dilution method. A stock solution of the extract (25, 50 and 100 µg/ml) in dimethyl formamide was prepared and graded quantities of the extract were incorporated in specified quantity of molten sterile agar (sabouraud dextrose agar medium for antifungal activity). A specified quantity of the medium (40-50°C) containing the extract was poured into a petridish to give a depth of 3-4 mm and allowed to solidify. Suspension of the microorganisms were prepared to contain approximately 105 cfu/ml and applied to plates with serially diluted extract in dimethyl formamide and incubated at 37°C for 24 hrs and 48 hrs respectively. The MIC was considered to be the lowest concentration of the test substance exhibiting no visible growth of fungi on the plate [7]

### Protein preparation:

Three dimensional structure of the Dihydrofolate reductase enzyme was retrieved from database using its id 1AI9. Protein was prepared by autodock tools. The ligand and crystallographic water molecules were removed from the protein, and the chemistry of the protein was corrected for missing hydrogen. Crystallographic disorders and unfilled valence atoms were corrected using alternate conformations and valence monitor options. Following the above steps of presentation, the protein was subjected to energy minimization by applying kollman charges [8].

### Ligand preparation:

The ligand molecule present in Mollugo cerviana was identified through literature search [9]. Three-dimensional structure of the colchicine, lupeol, qucertin phytocompounds was retrieved through pubchem text search and the structure was downloaded in .sdf format. The three-dimensional structure of phytocompound saved in .sdf format was converted to .pdb format using open babel 2.3.1. Ligands were prepared using MGL tools by adding hydrogen atom to check the valencies of the heavy atoms. Ligand was minimized by computing gasteiger charges and saved in PDBQT [10].

### Docking:

Docking program Autodock vina uses a grid-based method for energy evaluation of flexible ligand in complex with a rigid protein. Points on a 3D grid, are placed to cover the entire receptor. Docking was carried out using Autodock Vina with AMBER force field and Monte Carlo simulated annealing algorithm [11]. Throughout the docking studies the protein molecule was kept as rigid and drug molecules as flexible.

## Results and Discussion:

Aspergillus niger was found to be the most inhibited pathogen with a diameter of zone of inhibition from 33.45 mm. The IZD, it ranges between 15.61 mm to 33.45 mm. The extract was more active against Aspergillus niger with IZD of 33.45 mm and it has less activity against Candida albicans with IZD of 24.51 mm. Ketoconazole was used as standard against the fungal strains and the IZD ranges from 37.92 mm to 38.71 mm (Figure 1 - see PDF). The results showed that the MIC values against the tested strains ranges from 10.5 µg/ml - 15.5 µg/ml. Candida albicans had the highest MIC value of 15.5 µg/ml, while the lowest MIC of 10.5 µg/ml was shown by Aspergillus niger (Table 1 - see PDF). The above results clearly indicates that the phytochemicals present in the plants are a very good source of antifungal activity and proved to be very good alternatives for synthetic drugs. The extracts show a great activity against Aspergillus niger when compared to Candida albicans. The Plant has a very good antibacterial activity and this is proved in many previous experiments [12][13]. In the present study it is proved that they also exhibit a good antifungal activity. It has been reported that traditional medicine from plant extracts would contribute to solve 80% of the health problems around the world. Several papers and reviews have been published on the occurrence of antifungal compounds in plant. Hence further studies are required for the identification of the bioactive compound responsible for the antifungal activity. The chosen compounds colchicine, lupeol, qucertin has a very good affinity towards DHFR enzyme and believed to be possess a bioactive drug molecule.

## Docking studies:

Docking was performed with the compounds lupeol, qucertin and colchicine against DHFR. DHFR enzyme is a drug target for most of the fungal infection. Targeting this enzyme will leads to thymine less death to fungi. Docking is a computational method attempt to predict the noncovalent interaction between macromolecule and the drug. In the present study docking studies was performed by using Auto dock vina. Binding affinity value was used to identify how the compound strongly binds to the target protein. Results of this docking studies showed that colchicines showed the good binding affinity (-8.3 kcal/ mol) with DHFR compared to other two compounds (qucertin (-7.9kcal/mol and lupeol -6.3kcal/mol) and also its formed two hydrogen bond interaction with ALA-11 and GLU-32 residues. The docking results of all compounds are shown Table 2(see PDF). The results of interaction between DHFR receptor with the phytocompound are shown in Figure 2(see PDF). The green dotted line denotes the hydrogen bond. All the amino acid residues which involved in molecular interactions are displayed as lines and the ligand is displayed as sticks.

## Conclusion:

We report the molecular docking analysis of bioactive compounds in Mollugo cerviana (L.) SER with the DHFR protein target for antifungal activity against Aspergillus Niger and Candida albicans and it is proved that this plant extracts have good inhibitory activity against Aspergillus niger and colchicine was found to have a greater binding affinity against DHFR enzyme.
